# Impact of Polyvinylpyrrolidone-Vinyl Acetate Copolymer and Sodium Starch Glycolate Excipients on Phenolic Extraction from Red Clover: Enhancing Biological Activity and Antioxidant Potential

**DOI:** 10.3390/pharmaceutics16030399

**Published:** 2024-03-14

**Authors:** Jurga Andreja Kazlauskaite, Mindaugas Marksa, Jurga Bernatoniene

**Affiliations:** 1 Department of Drug Technology and Social Pharmacy, Lithuanian University of Health Sciences, 44307 Kaunas, Lithuania; jurga.andreja.kazlauskaite@lsmu.lt; 2 Institute of Pharmaceutical Technologies, Lithuanian University of Health Sciences, 44307 Kaunas, Lithuania; 3 Department of Analytical and Toxicological Chemistry, Lithuanian University of Health Sciences, 44307 Kaunas, Lithuania; mindaugas.marksa@lsmu.lt

**Keywords:** excipients, red clover, *Trifolium pratense*, isoflavones, polyphenols, polyphenolic activity, extraction

## Abstract

Adding certain excipients during the extraction process can enhance the concentration of target compounds, leading to potentially increased biological properties of the plant extract. This study explores the impact of PVP/VAC and SSG excipients on red clover bud extracts, aiming to enhance their concentration of target compounds and, consequently, their biological properties. The antioxidative potential was evaluated using DPPH, ABTS, and FRAP methods, and the chemical profile was determined using mass spectrometry. Antibacterial activity against various strains was determined through the minimal inhibitory concentration (MIC) method. The results revealed that the excipient-enriched samples exhibited significantly elevated antioxidant activities as well as phenolic and flavonoid contents compared to control samples. Notably, sample V1E3 demonstrated the highest antioxidant potential, with 52.48 ± 0.24 mg GAE/g dw (phenolic content), 463 ± 6.46 μg TE/g dw (ABTS), 12.81 ± 0.05 μg TE/g dw (DPPH), and 29.04 ± 1.16 mg TE/g dw (post-column ABTS). The highest flavonoid content was found in the S1E3 sample—24.25 ± 0.17 mg RU/g dw. Despite the increased antioxidant potential, no significant variance in antimicrobial activity was noted between the test samples and controls. This implies that excipients may hold the potential to enhance the biological properties of red clover extracts for pharmaceutical applications. These findings contribute valuable insights into optimizing extraction processes for improved functionality and application of plant-derived compounds in therapeutic formulations.

## 1. Introduction

Polyphenolic compounds in plant extracts are known for their radical scavenging capacity and antioxidant activity, with flavonoids being one of the phenol groups that possess a wide range of biochemical and pharmacological actions such as anticarcinogenic, antiviral, antimicrobial, antithrombotic, anti-inflammatory, and antimutagenic activities [1]. Flavonoids act as free radical scavengers, making them effective antioxidants in oils, fats, and emulsions [2]. Isoflavones, especially genistein and daidzein, like flavonoids, also possess antioxidant activities and are found mainly in legumes. In the past decade, extensive research has focused on various plant extracts that are rich in isoflavones due to their potential protective effects against a range of disorders. These include cardiovascular disease, cancer, hyperlipidemia, menopause symptoms, osteoporosis, and diabetes. Additionally, these extracts exhibit diverse bioprotective properties such as antioxidant, antimutagenic, anticarcinogenic, and antiproliferative activities [3,4,5,6].

Nowadays, soybeans and soy-derived foods are a major source of isoflavonoids in human nutrition. However, due to the allergenic nature of many soy products and the difficulty in obtaining genetically modified organism-free soybeans, there is a need for alternative sources of isoflavonoids. Among the alternative sources of isoflavones is red clover (*Trifolium pratense* L.), which has recently received considerable interest as a potential source of health-enhancing isoflavones [7,8,9,10]. A study found that 36% of the total red clover extract was identified. Within this analysis, 22 compounds were identified. Notably, the extract consisted of 35.54% isoflavones, 1.11% flavonoids, and 0.06% pterocarpans, while coumarins and tyramine were detected in trace amounts (≤0.03%) [11]. It is of the utmost importance to study the antibacterial and antioxidant properties of red clover flower samples’ phenolic components, particularly isoflavones, which are known to protect against several conditions. With antibiotic resistance becoming a global concern, plant extracts and phytochemicals with known antimicrobial properties are significant for treatment and prevention [12].

Plants accumulate a multitude of phenolic compounds that may interact synergistically or antagonistically. Phenolic compounds exhibit synergistic or antagonistic interactions due to their diverse structures and functional groups. Similar structures and functional groups lead to cooperative effects, especially when compounds target the same receptors, enhancing their overall effects; e.g., isoflavone phytoestrogens have estrogen receptor binding abilities [13]. Conversely, antagonistic interactions may occur when compounds compete for binding sites. Phenolic compounds, known for their antioxidant properties, can demonstrate synergy in combined effects, while antagonistic interactions may result from interference with their antioxidant activity. These compounds also modulate enzymatic activity, showing synergy in collectively enhancing or inhibiting enzyme pathways. However, antagonistic interactions may arise if compounds interfere with each other’s impact on enzymatic processes. Additionally, phenolic compounds can disrupt or enhance cellular metabolic pathways, with synergistic or antagonistic effects depending on their collective impact [14,15,16].

However, the extraction conditions could potentially degrade these compounds, or the properties of the solvent employed might hinder their efficient extraction from the plant. Using excipients in extraction media can help increase the yields of target compounds depending on their mechanism of action.

Previous research has shown that the use of sodium starch glycolate (SSG) and polyvinylpyrrolidone-vinyl acetate copolymer (PVP/VAC) increases the yield of the isoflavones daidzein and genistein; therefore, the excipient’s effect was determined by comparing the antioxidant potential and microbial activity of the red clover extracts [17]. This was due to the fact that modern carriers with a large surface area and high absorption capacity can help improve the oral bioavailability of poorly water-soluble drugs. Incorporating higher doses of these compounds into liquid–solid systems makes them more soluble and can improve bioavailability.

SSG, a commonly used super-disintegrant, accelerates the disintegration and dissolution of solid dosage forms, being FDA-approved for both prescription and over-the-counter medications [18,19]. SSG is produced through the chemical modification of starch, specifically carboxymethylation, for heightened hydrophilicity and cross-linking to decrease solubility [20].

SSG functions as a super-disintegrant by swiftly absorbing water, swelling, and exerting mechanical stress on the tablet matrix, facilitating rapid disintegration. Its swelling properties improve water penetration, aiding active ingredient dissolution. Optimization involves considerations such as particle size, degree of substitution, and concentration, while factors like pH, temperature, and compression force offer insights into disintegration and dissolution, with higher pH values and temperatures enhancing its effectiveness [19,21].Thus, reflux (high temperatures) and ultrasound aid in faster solvent dissipation.

PVP/VAC is a commonly used excipient in the pharmaceutical industry. It is soluble in both water and alcohol and is valued for its ability to improve the solubility of poorly soluble compounds by reducing particle size and increasing surface area for dissolution [22]. The copolymer can serve as a carrier or stabilizer for drug molecules, averting aggregation and maintaining an amorphous state that is conducive to dissolution and absorption in the body [23]. Daily dosage recommendations stand at 100 mg per tablet (10% in a 1000 mg tablet) for high consumers, with anticipated exposures of 200 mg PVP/VAC/day for children and 700 mg PVP/VAC for adults [22]. Comprising 1-vinyl-2-pyrrolidone and vinyl acetate in a 6:4 mass ratio, the current literature on PVP/VAC indicates solid dispersion preparation as a predominant approach to enhance the solubility and bioavailability of water-insoluble drugs [24,25,26].

By conducting a comparative analysis with a control group lacking these excipients, we investigated whether the inclusion of PVP/VAC and SSG could enhance the biological and microbial activity of the plant extract. This is the first study to investigate the effect of PVP/VAC and SSG excipients on plant biological activity. Consequently, it contributes valuable insights to the scientific understanding of the role played by these excipients in the development of red clover-based medicinal applications.

Therefore, this study aimed to determine the excipient’s effect by comparing the antioxidant potential and microbial activity of the red clover extracts prepared using excipients and without them.

## 2. Materials and Methods

### 2.1. Plant Material and Reagents

Red clover samples were harvested in Laičiai, Kupiškis district, Lithuania, on 31 July 2021. Prior to extraction, the flower buds were ground to a fine powder using an Ultra-Centrifugal Mill ZM 200 (Retsch, Haan, Germany). The grinding parameters included a force of 4025 g and a 0.5 mm trapezoid-hole sieve. Subsequently, the moisture content of the milled red clover was assessed using an MLB apparatus (KERN & Sohn GmbH, Balingen, Germany).

In this study, purified water was prepared using GFL2004 (GFL, Burgwedel, Germany), while deionized water was obtained from Millipore, SimPak 1 (Merck, Darmstadt, Germany). The excipients utilized in the samples included a vinylpyrrolidone-vinyl acetate copolymer (PVP/VAC) (Molecular weight~50,000) sourced from Yigitoglu Kimya, Istanbul, Turkey, and sodium starch glycolate (SSG) (Molecular weight~515.6862) acquired from Sigma-Aldrich, Taufkirchen, Germany.

To obtain standardized isoflavone samples, genistein, genistin, daidzein, and daidzin (Sigma Aldrich, Steinheim, Germany) were employed. A mixture of 2.21 mg of genistein and 2.18 mg of daidzein was prepared in 96% ethanol, while 3.30 mg of genistin and 3.03 mg of daidzin were mixed with 1 mL of DMSO and then diluted up to 10 mL using 96% ethanol.

Various reagents were employed in the experiment, including 96% ethanol (Vilniaus Degtinė, Vilnius, Lithuania), 2,2′-azino-bis(3-ethylbenzothiazoline-6-sulfonic acid) (ABTS), 2,4,6-Tris(2-pyridyl)-s-triazine (TPTZ), aluminum chloride, hexaethylenetetramine, dimethyl sulfoxide (DMSO), acetic acid, and Sabouraud dextrose agar (dehydrated) obtained from Sigma-Aldrich (Buchs, Switzerland). Additionally, potassium persulfate was procured from Alfa Aesar (Karlsruhe, Germany), while monosodium phosphate, ferrous sulfate heptahydrate, saline phosphate buffer, and hydrogen peroxide were sourced from Sigma Aldrich (Schnelldorf, Germany). Furthermore, disodium hydrogen phosphate was obtained from Merck (Darmstadt, Germany), and 2,2-diphenyl-1-picrylhydrazyl radical (DPPH) and Mueller-Hinton Agar (BBL, Baltimore, MD, USA) were also utilized.

### 2.2. Preparation of Red Clover Extracts and Sample Coding System

The extraction procedures followed the methodology outlined by Kazlauskaite et al. in a previous study [17]. Two extraction methods were employed: ultrasound combined with thermal hydrolysis, with varying ultrasound processing times, and heat-reflux extraction.

For the heat-reflux extraction, dried and milled flower heads were combined with solvent (either 50% ethanol *v*/*v* or purified water) in a round-bottom flask. The sample was refluxed in a sand bath at a solvent boiling temperature for 1 h and then cooled to room temperature. Following cooling, the sample underwent centrifugation, decantation, and filtration through PVDF syringe filters (pore size 0.22 µm, Frisenette, Knebel, Denmark).

Ultrasound-assisted extraction with thermal hydrolysis involved the use of an ultrasound bath (38 kHz) (Grant Instruments™, XUB12 Digital, Cambridge, UK) and a procedure similar to the heat-reflux method. The extraction involved macerating dried and milled flower heads in a solvent. Different conditions were applied, including the solvent type (50% *v*/*v* ethanol and purified water) and extraction time (10 or 30 min), with a processing temperature of 40 ± 2 °C. After processing, the sample was transferred to a round-bottom flask and refluxed in a sand bath for 1 h. Subsequently, the supernatant was treated as in the heat-reflux method.

The main samples in the experiment were prepared with excipients, PVP/VAC and SSG under the same conditions as previously mentioned, using either heat-reflux or ultrasound-assisted extraction with thermal hydrolysis. Purified water or 50% ethanol (*v*/*v*) served as the solvent, and the excipient was added to the extraction mixture along with the plant material. The PVP/VAC concentrations in the samples ranged from 1–5% (*w*/*v* in water) and 1% (in ethanol), while the SSG concentration was maintained at 1% (*w*/*v* in water and ethanol). Although the SSG concentration was increased to 5%, the resulting samples were too viscous for experimentation. Notably, all the excipients were removed from the extract during filtration, ensuring the absence of PVP/VAC or SSG in the final products.

The sample names comprised 4 characters: the first being a letter indicating the excipient (V for PVP/VAC and S for SSG), followed by the excipient concentration (V1 or V5 for PVP/VAC and S1 for SSG). The last letter indicated the solvent used (E for ethanol and W for water), with the number indicating the extraction conditions: 1 for reflux alone, 2 for reflux combined with 10 min ultrasound processing, and 3 for reflux combined with 30 min ultrasound processing.

**Figure 1:**
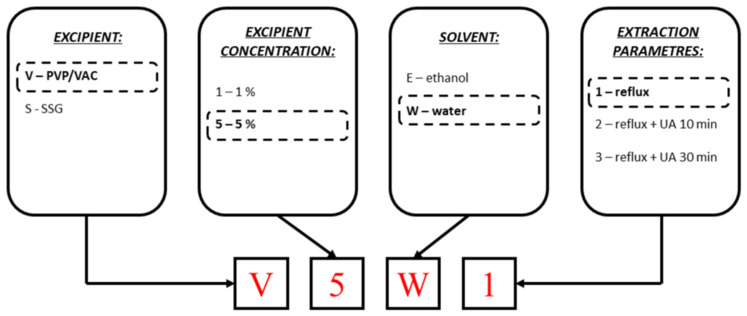
Schematic coding system of the samples.

Thus, if the sample is prepared with PVP/VAC 5% in water using reflux, the sample code will be V5W1. If these are control samples, there is no excipient letter in the code. Figure 1 shows a simplified coding scheme.

### 2.3. Determination of Total Phenolic and Flavonoid Contents

The determination of the total phenolic content was carried out according to Dewanto et al. [27]. Folin–Ciocalteu’s phenol reagent, along with 7% (*w*/*v*) sodium carbonate, was employed for the reaction. The absorbance was measured after 1 h using a spectrophotometer (765 nm) (Shimadzu UV-1800, Kyoto, Japan). The calibration curve used gallic acid (0–0.1 mg/g; y = 11.108; R^2^ = 0.9981). The results were reported as gallic acid equivalent per gram dry weight (mg GA/g dw).

For flavonoid content determination, the extract was mixed with 96% (*v*/*v* ethanol, 33% acetic acid, 10% aluminum chloride, and 5% hexaethylenetetramine solutions in a test tube. A spectrophotometric analysis was conducted after 30 min (475 nm) using a spectrophotometer (Shimadzu UV-1800, Kyoto, Japan). The flavonoid content was expressed as rutin equivalent mg RE/g dw (milligrams of standard antioxidant Rutin equivalents per gram of dry weight) and calculated using the following formula: TFC = C × Ve × F/M, where TFC represents the total flavonoid content in mg RE/g dw, C is the concentration of the standard used in mg/L, Ve is the volume of the solvent used in L, F is the dilution coefficient of the sample, and M is the mass of the sample in g. The calibration curve was established with rutin (0–0.5 mg/g; y = 5.0867; R^2^ = 0.9985).

### 2.4. Antioxidant Activity

#### 2.4.1. ABTS and DPPH Radical Scavenging Activity Assays

The ABTS radical was generated through the oxidation of ABTS with potassium persulfate [28]. The results were expressed as Trolox equivalent µg TE/g dw (micrograms of standard antioxidant Trolox equivalents per gram of dry weight). The calibration curve was obtained using Trolox (0–0.5 mg/g; y = 0.0001728x; R^2^ = 0.9832).

A DPPH working solution (0.1 mM in ethanol) was prepared, and the reaction procedure followed the method described in a study by Gómez-Alonso et al. [29]. The calibration curve was obtained using Trolox (0–0.016 mg/g; y = 0.00623x; R^2^ = 0.9923). The results were expressed as Trolox equivalent µg TE/g dw (micrograms of standard antioxidant Trolox equivalents per gram of dry weight).

#### 2.4.2. Ferric Reducing Antioxidant Power (FRAP)

The sample was mixed with FRAP reagent as described in a previous study [30]. The calibration curve was obtained using ferrous sulfate (0–1 mg/g; y = 2.6272; R^2^ = 0.9985). The results were expressed as ferrous sulfate equivalent mg FS/g dw (milligrams of standard ferrous sulfate equivalent per gram dry weight).

### 2.5. Antimicrobial Activity

The antimicrobial activity was assessed using the diffusion method on solid nutrient media, specifically Mueller–Hinton agar (BBL, Baltimore, MD, USA). Standard reference microorganisms from the ATCC (American Type Culture Collection) were employed to evaluate the antimicrobial efficacy of the tested extracts. Cultures of non-spore bacteria, including *Staphylococcus aureus* (ATCC 25923), *Staphylococcus epidermidis* (ATCC 12228), *Enterococcus faecalis* (ATCC 29212), *Escherichia coli* (ATCC 25922), *Klebsiella pneumoniae* (ATCC 13883), *Pseudomonas aeruginosa* (ATCC 27853), and *Proteus vulgaris* (ATCC 8427), were cultivated for 20–24 h at 35–37 °C on the agar.

Bacterial suspensions were prepared from these cultivated cultures in sterile physiological sodium chloride (0.9%) solution and standardised using a McFarland standard indicator. Standardisation was achieved when the indicator value reached 0.5, indicating that 1 mL of the bacterial suspension contained approximately 1.5 × 10^8^ cells of the microorganism.

Standard cultures of *Bacillus cereus* (ATCC 11778) spore bacteria were cultivated for 7 days at 35–37 °C on Mueller–Hinton agar. Following bacterial growth, the spore bacteria culture was carefully rinsed from the surface of the medium using a sterile physiological solution. The resulting suspension underwent a 30 min heat treatment at 70 °C and was subsequently diluted with physiological saline until the spore concentration reached 10 × 10^6^–100 × 10^6^ per 1 mL.

*Candida albicans* (ATCC 10231) fungus standard cultures were grown for 20 to 24 h at 30 °C over a span of 72 h on Sabouraud dextrose agar. A fungal suspension was prepared from these cultivated cultures in physiological saline and standardised using a McFarland standard.

Different concentrations of red clover extract were dispensed into Petri dishes. Subsequently, 5 mL of 45 °C liquid Mueller–Hinton agar was added to each sterile Petri dish and thoroughly mixed with the added sample volumes. Once the agar solidified, suspensions prepared from reference microorganisms were inoculated into segments within the Petri dishes. The bacterial cultures were then incubated with samples in a thermostat for 20–24 h at 35 °C, followed by an additional 24 h of storage at room temperature.

The antimicrobial activity of the red clover flowers’ ethanolic and aqueous extracts and antibiotics such as penicillin, ciprofloxacin, ampicillin, amoxicillin, and daptomycin were assessed.

### 2.6. HPLC–PDA Conditions for Isoflavone Quantification

Isoflavone quantification was conducted using the high-performance liquid chromatography with photodiode array detection (HPLC-PDA) method, following the exact procedures outlined in a study by Kazlauskaite et al. [31].

For the determination of the isoflavones daidzein, genistein, daidzin, and genistin, an ACE 5 C18 250 × 4.6 mm column (Advanced Chromatography Technologies, Aberdeen, Scotland) was used. The mobile phase of solvent A consisted of acetic acid, methanol, and deionised water (using the ratio of 1:10:89 *v*/*v*/*v*). Solvent B was made using acetic acid and methanol (1:99 *v*/*v*/*v*). The linear gradient elution profile was as follows: 80% A/20% B at 0 min; 30% A/70% B at 30 min; 90% A/10% B at 39 to 40 min. The flow rate was 1 mL/min, and the injection volume was 10 µL. The absorption was measured at 260 nm.

### 2.7. HPLC Post-Column Antioxidant Activity

The HPLC post-column method was carried out using ABTS reagent according to the methodology described by Marksa et al. [32].

Following the introduction of samples into the HPLC detection system, the mobile phase, along with the sample, traversed through the reaction loop to a mixing tee. Concurrently, a Gilson pump 305 (Middleton, WI, USA) delivered 0.5 mL/min of ABTS reagent solution. The HPLC-ABTS system’s reaction loop was maintained at a constant temperature of 50 °C. Upon interaction with ABTS, the antioxidants triggered a color change in the reagent, which was quantified using a Waters 2487 UV/VIS detector (Waters Corporation, Milford, MA, USA). The test solutions were detected at a wavelength of 650 nm, with the signal strength depicted as the peaks of negative active compounds.

The antioxidant activity of the extract compounds was assessed relative to the Trolox standard equivalent. A calibration curve was generated using a Trolox/ethanol solution at five dilutions within the 8.359–133.750 g/mL range, R^2^ = 0.999565.

### 2.8. Qualitative Analysis of Red Clover Extracts Using LC-MS

Following the reverse phase liquid chromatography (RP-LC) separation, electrospray ionisation (ESI) (in the negative and positive modes) and mass spectrometric (MS) analyses were performed to determine the qualitative profile of the red clover extract. The LC/MS system was composed of a Shimadzu Nexera X2 LC-30AD HPLC system (Shimadzu, Tokyo, Japan) equipped with an LCMS-2020 mass spectrometer (Shimadzu, Tokyo, Japan).

The chromatographic separation was performed using a YMC-Triart C18 (YMC Karasuma-Gojo, Kyoto, Japan) (150 mm × 3.0 mm, 3 μm) analytical column, and its temperature was 40 °C.

Mobile phase A consisted of 0.1% formic acid in the water, and mobile phase B consisted of 0.1% formic acid in acetonitrile. For each clover sample, 1 μL aliquot volumes were injected into the chromatographic column. The linear gradient elution profile was as follows: 10% B at 0.01 min; 10% B at 1.0 min; 50% B at 10 min; 70% B at 20 min; 90% B at 23 min; 90% B at 26 min; 10% B at 27 min; 10% B at 30 min. The HPLC was run at a flow rate of 0.4 mL/min.

The optimum ESI conditions were set as 350 °C for the interface temperature, 250 °C for the DL temperature, 400 °C for the heat block temperature, 1.5 L/min for the nebulising gas flow, and 10 L/min for the drying gas flow. The positive ion and negative ion measurements were performed while switching alternately between the positive and negative ionisation modes. The *m*/*z* ranges for the positive and negative modes were 50–1000, the scan speed was 15.000 μ/s, and a step size of 0.1 *m*/*z* was used.

The quantitative analysis of the identified compounds was conducted utilising hyperoside as a reference standard. The compounds’ concentrations were determined by applying a hyperoside calibration curve. The results were expressed as micrograms of hyperoside equivalent per milliliter (μg/mL). The calibration curve was prepared from a hyperoside solution at eighth dilutions in the range of 0.21–27.04 µg/mL, y = 22,498.4x + 2508.57, R^2^ = 0.9998.

### 2.9. Statistical Data Analysis

The data were analysed using SSPS version 20.0 (IBM Corporation, Armonk, NY, USA). All the antioxidant experiments were performed 4 times. The data are expressed as mean ± standard deviation (S.D.). The antimicrobial experiments were repeated 3 times. Comparisons between the three different measurements were made using Friedman and Wilcoxon tests. Comparisons between the two groups were made using the Mann–Whitney U test. The results were considered statistically significant at *p* < 0.05. Spearman’s correlations were performed at a significance level of 0.01 (two-tailed). The strength of association was judged based on the following scores: |r| ≥ 0.9—very strong; 0.7 ≤ |r| < 0.9—strong; 0.5 ≤ |r| < 0.7—moderate; 0.3 ≤ |r| < 0.5—weak; |r| < 0.3—very weak.

## 3. Results and Discussion

### 3.1. Total Phenolic and Flavonoid Content

The total phenolic and flavonoid content results were obtained by applying spectrophotometry. This technique allows for quantitative composition determination of groups of biologically active compounds. Additionally, PVP/VAC (1–5%) and SSG (1%) were incorporated into the samples to analyze and determine their total phenolic content, flavonoid content, and antioxidant activity. It is important to note that these excipients alone do not possess antioxidant properties [17].

The methodologies chosen for studying raw plant materials were selected to assess the variability of phenolic compounds and flavonoids in red clover extracts prepared using different excipients (SSG and PVP/VAC). The red clover extracts with excipients were prepared in 50% ethanol and 70 and 96% ethanol solutions. Following testing, the differences in the results among the solvents were not statistically significant. Consequently, the decision was made to use the ethanol solution with the lowest concentration (50%). A previous study guided the selection of the extraction parameters mentioned earlier [17].

**Figure 2:**
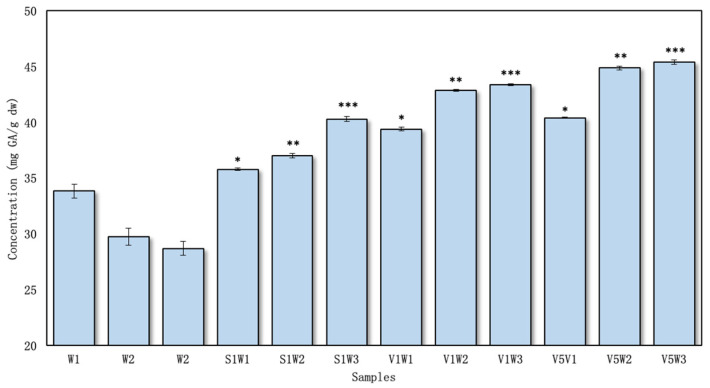
The total amount of phenolic compounds in the aqueous extracts, *n* = 4 (the codes of the samples are provided in Figure 1). * *p* < 0.05 vs. W1; ** *p* < 0.05 vs. W2; *** *p* < 0.05 vs. W3 (the samples were compared with their respective control groups).

**Figure 3:**
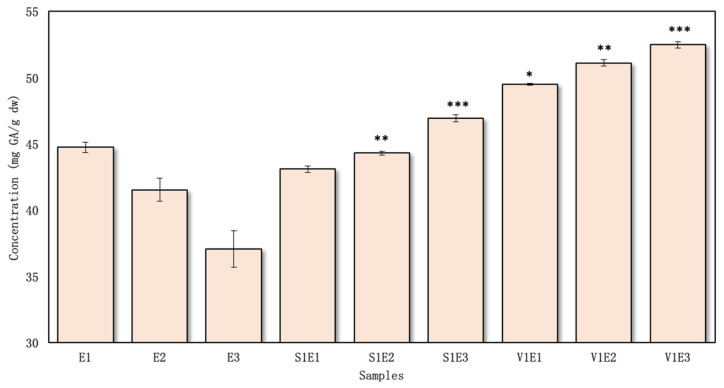
The total amount of phenolic compounds in ethanolic extracts, *n* = 4 (the codes of the samples are provided in Figure 1). * *p* < 0.05 vs. E1; ** *p* < 0.05 vs. E2; *** *p* < 0.05 vs. E3 (the samples were compared with their respective control groups).

The total amount of phenolic compounds in the red clover aqueous flower samples with excipients was determined to vary from 35.79 ± 0.12 to 45.38 ± 0.19 mg GAE/g dw (Figure 2), and in the ethanolic samples, it varied from 43.11 ± 0.24 to 52.48 ± 0.24 mg GAE/g dw (Figure 3).

Among the aqueous samples, V5W3 exhibited the highest concentration of phenolic compounds, measuring 45.38 ± 0.19 mg GAE/g dw. In contrast, the sample S1W1 displayed the lowest phenolic compound content, amounting to 35.79 ± 0.12 mg GAE/g dw (Figure 2).

Increasing the concentration of PVP/VAC excipient in the extraction media from 1% to 5% led to overall increases. However, a statistically significant difference (*p* < 0.05) was specifically observed in the samples prepared through a combination of reflux and ultrasound processing. Importantly, across all the samples prepared with excipients in both 50% ethanol and water solvents, there were statistically significant differences (*p* < 0.05) compared to the control samples.

The highest total phenolic content in the ethanolic extracts was determined for the sample V1E3 (52.48 ± 0.26 mg GAE/g dw), and lowest was found for S1E1—43.11 ± 0.24 mg GAE/g dw (Figure 3). PVP/VAC demonstrated a greater extraction efficacy than the SSG excipient, even without increasing the concentration from 1% to 5%. Specifically, when employing PVP/VAC, there was an average increase of 11% in the amount of phenolic compounds compared to using SSG.

In research by Küçükboyaci et al., the total phenolic content was determined to be 52.30 ± 1.20 mg GAE/g [33]. This yield is higher than that found in our study without and with excipients. Esmaeili et al. investigated in vivo grown red clover, which varied from 27.57 to 46.88 mg GAE/g dw. These results are similar to the results obtained in this study without using toxic solvents but using green extraction with excipients [34]. However, the study by Küçükboyaci et al. used methanol for extraction, an effective solvent based on medicinal plant research [33]. The second study used different organic solvents (methanol, ethyl acetate, chloroform). Notably, the results obtained using ethyl acetate were found to be ineffective. While all the solvents used in the studies are capable of extracting both polar and non-polar secondary metabolites, it is important to highlight that methanol and chloroform present challenges due to their toxicity, rendering them unsuitable for studies involving organisms and animal models. The procedural step of solvent evaporation, although implemented, may still leave trace amounts behind [35]. There is also the possibility that differences in the composition of phenolic compounds can be influenced by biotic and abiotic factors, plant parts, harvesting time, and inaccuracies in taxonomic identification and chemical analysis [36,37]. It is not easy to compare chemical data because there is a great deal of inconsistency, but it can provide an overall picture of a species’ chemical profile.

Using both excipients (PVP/VAC and SSG) in ethanol/water extraction was more effective than extraction using the solvent alone. However, regardless of the effectiveness of both excipients, the use of PVP/VAC in the extraction helped to extract higher amounts of phenolic compounds in both water and ethanol (Figure 2 and Figure 3).

As solutions cool down, both excipients gradually settle into sediments. The sedimentation of these excipients, rather than being a hindrance, present an opportunity for resource optimization. The settled excipients can be effectively recovered and reused in extraction processes. This sustainable approach not only minimizes waste but also contributes to cost-effectiveness in various applications. Industries can enhance their efficiency and reduce their environmental impact by harnessing the recoverable excipients.

The phytochemical profile of red clover includes a wide range of polyphenolic compounds, such as numerous flavonoids (including isoflavones) [38]. Most flavonoids exist naturally as glycosides. The presence of sugars and hydroxyl groups makes them water soluble, while the presence of methyl groups makes them lipophilic.

**Figure 4:**
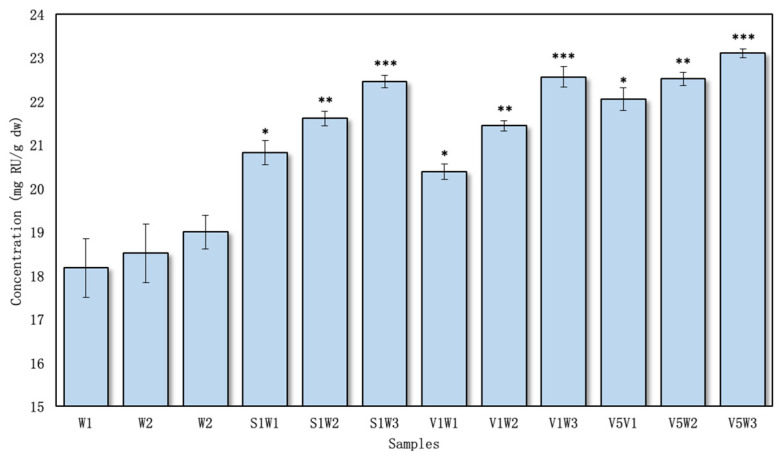
The total amount of flavonoids in aqueous extracts, *n* = 4 (the codes of the samples are provided in Figure 1). * *p* < 0.05 vs. W1; ** *p* < 0.05 vs. W2; *** *p* < 0.05 vs. W3 (the samples were compared with their respective control groups).

**Figure 5:**
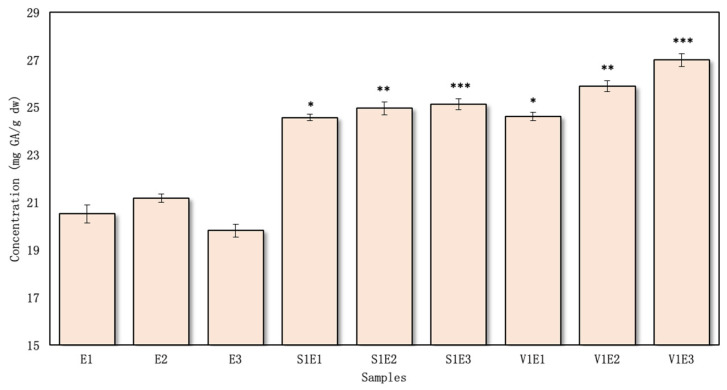
The total amount of flavonoids in ethanolic extracts, *n* = 4 (the codes of the samples are provided in Figure 1). * *p* < 0.05 vs. E1; ** *p* < 0.05 vs. E2; *** *p* < 0.05 vs. E3 (the samples were compared with their respective control groups).

The content of total flavonoids was expressed as milligrams of rutin equivalents per gram of dry sample, ranging from 20.38 ± 0.23 to 23.10 ± 0.11 mg RU/g dw in the aqueous test extracts (Figure 4) and 24.57 ± 0.13 to 26.98 ± 0.28 mg RU/g dw in the ethanolic extracts with excipients (Figure 5).

The highest amount of flavonoids from the aqueous samples was found in V5W3 (23.10 ± 0.11 mg RU/g dw) (Figure 4). The lowest content of flavonoid compounds was found in the S1W1 and V1W1 samples (20.82 ± 0.27 and 20.38 ± 0.23 mg GAE/g dw, respectively). All the samples prepared using the excipients in water yielded statistically significant differences (*p* < 0.05) compared to the controls.

Observing the results, it was determined that when using the PVP/VAC excipient, the lowest outcomes were observed with reflux alone, but an increase in flavonoid yield occurred when ultrasound was incorporated and the processing time was extended from 10 to 30 min. Similarly, a parallel pattern of increased flavonoid yield was observed when using the SSG excipient in the extraction process.

The investigation revealed a direct correlation between the concentration of PVP/VAC and the increase in flavonoid levels, specifically from 1% to 5%. A significant (*p* < 0.05) rise in flavonoid yield was noted when comparing samples with PVP/VAC concentrations of 1% and 5%. While a comparable effect was observed when comparing the results from both excipients, it is noteworthy that adjusting the PVP/VAC concentration in the extraction media demonstrated the potential for further increases in flavonoid concentrations.

Using ethanol as a solvent, the total flavonoid amount was the highest in the sample V1E3 prepared with PVP/VAC excipient (1%) (26.98 ± 0.28 mg RU/g dw) (Figure 5). The lowest flavonoid amount was found in the S1E1 and V1E1 samples (24.57 ± 0.13 and 24.61 ± 0.17 mg RU/g dw). The results obtained using ethanol and excipients were better than those obtained using water. Still, the same trends remained: the extracts prepared with PVP/VAC and SSG using only reflux yielded the lowest flavonoid concentrations, but when combining this method with ultrasound and increasing the processing time, the concentrations increased. The results were statistically significantly higher than the controls prepared without excipients (*p* < 0.05).

### 3.2. Excipients’ Influence on Radical Scavenging Activity

Due to the fact that the electron reduction potential of phenolic radicals is lower than that of oxygen radicals and that phenoxyl radicals are less reactive than oxygen radicals, phenolic compounds are effective scavengers of oxygen radicals [39]. Many of polyphenolics’ biological functions have been associated with their ability to scavenge and neutralize free radicals and their antioxidant activity, including antibacterial, anti-inflammatory, antiallergic, hepatoprotective, antithrombotic, antiviral, anticarcinogenic, and vasodilator effects [40,41].

Many factors, such as the plant growth environment, the composition of the extract, and the test system, influence the antioxidant capacity of the extract from the plant. Therefore, it cannot be fully described using one single method to obtain objective results. A reliable antioxidant assay protocol requires measuring more than one property using several different methods, since most natural antioxidants are multifunctional [42,43]. The antioxidant activity of phenolic-rich plant materials correlates with their antioxidant capacity, which depends on the structure of the compounds.

The antioxidant activity was evaluated using ABTS and DPPH in vitro techniques that have different experimental conditions but belong to single electron transfer-based assays. The results in the samples with excipients investigated using the DPPH method varied from 10.17 ± 0.02 to 11.25 ± 0.11 µg TE/g dw (Table 1) in the aqueous samples and 11.26 ± 0.06 to 12.81 ± 0.05 µg TE/g dw (Table 1) in the ethanolic samples.

Analyzing the antioxidant results of the aqueous samples obtained using the DPPH method, it was determined that the highest antioxidant activity was found in the V5W3 sample (11.25 ± 0.11 µg TE/g dw) that was prepared using an enhanced amount (5%) of PVP/VAC. The lowest activity was determined in the sample S1W3 (10.17 ± 0.08 µg TE/g dw). All the aqueous samples were statistically significantly different (*p* < 0.05) compared with controls that were prepared without the excipients (Table 1). It was determined that the samples prepared using PVP/VAC had antioxidant activities that were significantly (*p* < 0.05) higher than those prepared using the SSG excipient.

The highest antioxidant activity in the ethanolic samples was found in the V1E3 sample (12.81 ± 0.05 µg TE/g dw), and the lowest was found in the S1E2 sample (11.26 ± 0.06 µg TE/g dw) (Table 1). All the samples were statistically significantly different compared with the controls. In ethanol, using the SSG excipient, ultrasound processing for 10 min, and reflux, the antioxidant activity was lower than when using reflux alone, but the activity increased with increasing processing times. However, using PVP/VAC in extraction reflux resulted in the lowest antioxidant activity.

Limited literature is available on the assessment of phenolic compounds and antioxidant activity in red clover flowers. Most studies focus on extracts from either the entire plant or, specifically, the leaves. In research by Jakubczyk et al., red clover flowers were extracted using a Soxhlet apparatus and 96% ethanol as a solvent [44]. The determined antioxidant activity (DPPH) of the red clover extract was lower compared with the results in this study by approximately 30% (controls) and 33–39% compared with samples prepared using the excipients. In other research by Horvat et al., extracts were prepared from red clover leaves of different breeding populations [45]. The results showed that on average, DPPH inhibition was 18% lower compared to our controls and 24–27% lower compared with the samples prepared using the excipients. The right extraction method of phenolic compounds can increase antioxidant activity, and the right excipient can also increase antioxidant potential from 3 to 9%.

An ABTS assay offers the advantages of simplicity and speed, and it is a well-established method for estimating the antioxidant activity of test samples [46]. The radical scavenging ability measured using the ABTS assay is given in Table 1 and expressed as for the DPPH assay. The ABTS radical activity followed a similar pattern to the DPPH assay in both the aqueous and ethanolic extracts with excipients. Using the ABTS method, the results varied from 383.63 ± 7.73 to 419.29 ± 7.00 µg TE/g dw (Table 1) in the aqueous samples and 425.04 ± 4.38 to 463.92 ± 6.46 µg TE/g dw (Table 1) in the ethanolic samples.

The lowest antioxidant activity in the aqueous samples was detected in sample S1W2 (383.63 ± 7.73 µg TE/g dw) prepared with SSG excipient using reflux and ultrasound processing for 10 min. Among samples prepared using SSG antioxidant activity, adding ultrasound for the extraction decreased, but prolonging the time to 30 min (S1W3 sample, 388.60 ± 6.40 µg TE/g dw) (Table 1) increased the activity. However, it was still lower than when using reflux alone (S1W1 sample, 389.06 ± 6.45 µg TE/g dw). The highest antioxidant activity was determined in the V5W3 extract (419.29 ± 7.00 µg TE/g dw) that was prepared using 5% PVP/VAC and reflux combined with 30 min of ultrasound processing. This result was significantly higher (*p* < 0.05) than all the results from the aqueous extracts. However, even when using only 1% PVP/VAC, the antioxidant activity was significantly higher than when using SSG (Table 1).

The highest antioxidant activity in the ethanolic samples was determined in sample V1E3 (463.92 ± 6.46 µg TE/g dw). The lowest activity was found in the S1E2 (425.04 ± 4.38 µg TE/g dw) sample. Tendencies like those in the aqueous extracts were also observed in the ethanolic samples. All the results were statistically significantly higher compared to the controls.

Both the ABTS and DPPH methods showed similar results, which demonstrate that the results are reproducible and that these methods are complementary. The extracts prepared in ethanol were more effective, and when using ethanol with PVP/VAC, the antioxidant activity increased by approximately (not including the results obtained using 5% PVP/VAC) 14% (compared to aqueous extracts). Using SSG excipient in ethanol, the antioxidant activity increased by 10%.

Correlation analyses were performed to observe the relationship between the antioxidant activity and the chemical composition of the extracts for DPPH and ABTS, as well as the two major groups of compounds—the total amount of phenolic compounds and flavonoids. The results show a positive correlation between the total phenolic content of all the extracts (controls and test samples) and the antioxidant capacity measured using DPPH (r = 0.917 in ethanolic extracts, r = 0.939 in aqueous extracts) (*p* < 0.01) as well as ABTS (r = 0.932 ethanolic samples, r = 0.951 aqueous samples) (*p* < 0.01). This confirms that the phenolic compounds are the primary source of inherent antioxidant activity, which has additionally been stated in other publications [47,48].

Flavonoids also showed a positive correlation with both radical scavenging methods: r = 0.933 in ethanol, r = 0.753 in water (*p* < 0.01) (DPPH); r = 0.899 in ethanol, r = 0.832 in water (*p* < 0.01) (ABTS). The correlations were higher in the ethanol samples than in water because flavonoids are poorly soluble [49]. Nevertheless, excipient use increases flavonoid solubility using both excipients in water and ethanol.

The results obtained using both antioxidant activity methods, ABTS and DPPH, were in agreement, which was also reflected in the correlation coefficients of r = 0.953 for the aqueous samples and r = 0.982 for the ethanolic samples.

### 3.3. Reducing Power Activity of Extracts Prepared Using Excipients

**Figure 6:**
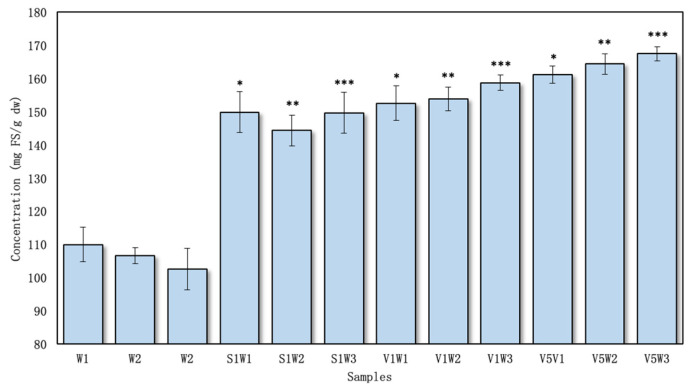
Antioxidant activity of aqueous samples using the FRAP method, *n* = 4 (the codes of the samples are provided in Figure 1). * *p* < 0.05 vs. W1; ** *p* < 0.05 vs. W2; *** *p* < 0.05 vs. W3 (the samples were compared with their respective control groups).

**Figure 7:**
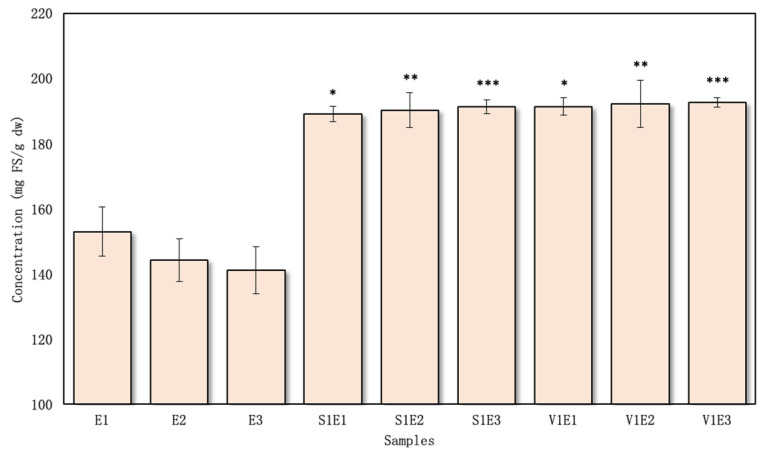
Antioxidant activity of ethanolic samples using the FRAP method, *n* = 4 (the codes of the samples are provided in Figure 1). * *p* < 0.05 vs. E1; ** *p* < 0.05 vs. E2; *** *p* < 0.05 vs. E3 (the samples were compared with their respective control groups).

The FRAP method is a simple, inexpensive, reproducible, and sensitive method to assess antioxidant power [46]. The obtained results were expressed as ferrous sulfate equivalent mg FS/g dw. The FRAP values of the studied extracts varied from 144.33 ± 4.60 to 167.45 ± 2.06 mg FS/g dw in the aqueous samples (Figure 6) and 189.03 ± 2.35 to 192.49 ± 1.46 mg FS/g dw in the ethanolic samples (Figure 7).

The highest value obtained using the FRAP method for the aqueous samples was found for the V5W3 sample—167.45 ± 2.06 mg FS/g dw (Figure 6), while the lowest value was noted in S1W2 at 144.33 ± 4.60 mg FS/g dw. The outcomes obtained using the FRAP method were consistent with those obtained using the ABTS and DPPH methods. Notably, all the test samples exhibited statistical superiority over the controls (*p* < 0.05). However, it is important to highlight that the results with PVP/VAC were significantly higher than those with SSG only when the former was used at a 5% concentration.

The antioxidant activity in the ethanolic test samples was very similar (Figure 7). All the samples had statistically higher activities than the controls, but no significant difference existed between the samples prepared using PVP/VAC and SSG. Nevertheless, the results obtained in this study were higher than those of Zawislak et al., who used different solvents for the extracts and different red clover drying conditions [50].

The previously presented results show a positive correlation between the total phenolic content and the ABTS/DPPH methods. These results were confirmed by the reducing power activity method FRAP, which also showed a positive correlation between the total amount of phenolic compounds: r = 0.950 (*p* < 0.01) in ethanol and r = 0.944 (*p* < 0.01) in the aqueous samples. The same correlation was observed between flavonoids and the FRAP method, but it was weaker in the aqueous samples r = 0.804 (*p* < 0.01). In the ethanolic samples, r = 0.950 (*p* < 0.01).

### 3.4. Phenolic Compounds’ Antioxidant Potential

Studies have shown that red clover contains the following isoflavones: genistein, daidzein, biochanin A, and formononetin [7,51]. Isoflavonoids have a variety of bioprotective effects, including antioxidant, antimutagenic, anticarcinogenic, and antiproliferative activities. In a previous study by Kazlauskaite et al. [17], the concentrations of the isoflavonoids daidzein and genistein, depending on the extraction conditions, were determined in the red clover extracts with PVP/VAC and SSG excipients. In this study, we used a mixture of the isoflavones daidzein, genistein, daidzin, and genistin and determined the antioxidant activity of the known sample. The preparation of the isoflavones mix from standards is described in Section 2.1—“Plant Material and Reagents”.

The obtained concentrations of isoflavones based on the HPLC analysis were as follows: genistein 97.29 ± 3.89 μg/g, daidzein 95.74 ± 3.83 μg/g, genistin 125.41 ± 5.01 μg/g, and daidzin 139.45 ± 5.57 μg/g. These concentrations were used to determine the antioxidant activity (total content of these isoflavones: 457.91 ± 18.31 μg/g).

In this study, the total amount of phenolic compounds, flavonoid content, and antioxidant activity of the samples were determined using the ABTS, DPPH, and FRAP methods. The total phenolic content was 7.55 ± 0.16 mg GA/g dw, and the determined flavonoid content was 12.77 ± 1.24 mg RU/g dw. We determined that antioxidant activities of the isoflavones samples were relatively low: 1.93 ± 0.05 µg TE/g dw (DPPH), 74.25 ± 0.65 µg TE/g dw (ABTS), and 24.42 ± 1.62 mg FS/g dw. Although glycosides (daidzin, genistin) are inactive forms, studies have shown that they have a similar antioxidant activity to aglycones in vitro [52,53].

A similar total concentration of isoflavones was found in sample S1E1 (464.80 μg/g: genistein 187.10 μg/g; daidzein 216.67 μg/g; genistin 61.03 μg/g; and daidzin 0 μg/g). Even though the isoflavone contents were similar, as mentioned before, the glycosides’ antioxidant action in vitro showed the same results. The results showed that the extract sample had 6 times more phenolic compounds but only 1.92 times more flavonoids. Though phenolic acids have been studied both in vitro and in vivo for their antioxidant properties, the mechanisms of their action remain unclear and/or undefined. An essential factor that should be considered is their mutual interactions, which can be synergistic, antagonistic, or additive (no interaction). These interactions were investigated by several studies using a variety of antioxidant assays, and both synergistic and antagonistic interactions were confirmed [15,54]. Although the antioxidant responses of the isoflavones tested in this study were small, they may act synergistically with other compounds in the extract, thus increasing the response of the whole product. Various compounds were investigated to act in synergy with daidzein or genistein [55,56,57,58]. The use of the red clover extract as a source of phenolics, including isoflavones, seems to be promising due to the presence of several molecules that can act in synergy with each other, promoting and enhancing antioxidant capability.

### 3.5. Post-Column ABTS Antioxidant Assay and RP-LC/PDA/MS Qualitative Analysis

Additionally, the best results were obtained for the samples that were investigated using the post-column ABTS method. The highest antioxidant activity using the post-column ABTS method in the aqueous samples was V1W3 20.90 ± 0.83 mg TE/g dw, and in the ethanolic samples, it was V1E3—29.04 ± 1.16 mg TE/g dw (Table 2). All the samples had statistically higher values compared with the controls prepared using the same method.

**Figure 8:**
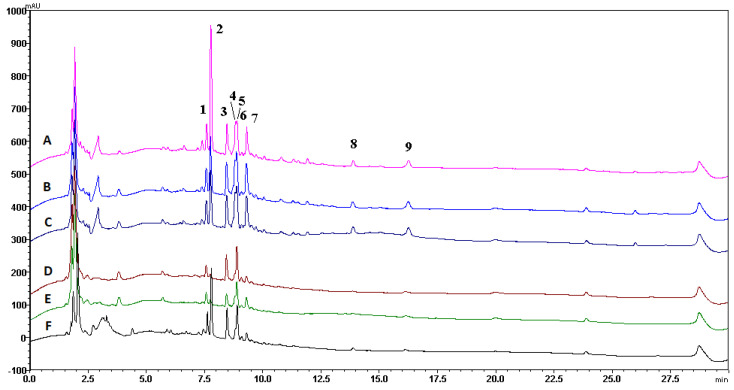
Profiles of control and test samples: A—V1E3 sample profile; B—S1E3 sample profile; C—E3 sample profile; D—S1W3 sample profile; E—W3 sample profile; F—V1W3 sample profile. The wavelength used to determine the compounds was 330 nm.

An LC-MS analysis of all the post-column samples was performed, and the determined compounds were compared to published red clover profiles. As a result, nine compounds were identified in the red clover extracts with and without the excipients (Figure 8A–F).

It was determined that peak 1 (*m*/*z* 295.0 [M-H]^−^) was caffeoylmalic acid, peak 2 (*m*/*z* 358.0 [M-H]^−^) was cis-clovamide, peak 3 (*m*/*z* 463.1 [M-H]^−^) was hyperoside, peak 4 (*m*/*z* 505.1 [M-H]^−^) was quercetin-O-hexoside-acetate, peak 5 (*m*/*z* 447 [M-H]^−^) was kaempferol-O-hexoside, peak 6 (*m*/*z* 477.1 [M-H]^−^) was methylquercetin-O-hexoside, peak 7 (*m*/*z* 489.1 [M-H]^−^) was ellagic acetyl rhaminoside, peak 8 (*m*/*z* 267 [M-H]^−^) was formononetin, and peak 9 (*m*/*z* 283 [M-H]^−^) was biochanin A [38,59] (Figure 8).

Many of the detected compounds like hyperoside, clovamide, and caffeomalic acid contribute to stress tolerance in numerous plant species due to their high antioxidant activity, increased cell viability, and antimicrobial activity [60,61,62]. Comparing the aqueous samples with the ethanolic extracts prepared using water demonstrated that the former had a narrower compound profile. Formononetin and biochanin A were not found.

The V1E3 sample, prepared using the PVP/VAC excipient, exhibited the highest antioxidant activity. However, the total concentration of identified compounds was higher in the S1E3 sample (Table 3). This phenomenon can be attributed to variations in the antioxidant properties of the compounds present. Consequently, compounds with higher concentrations in S1E3 displayed lower antioxidant capacities.

The lowest concentrations of the identified compounds were found in the aqueous samples as opposed to the ethanolic ones. Despite the samples prepared using PVP/VAC exhibiting better biological properties, the determined compounds’ concentrations were lower than those prepared with SSG, ethanolic, and aqueous solutions. However, the control samples showed very minimal concentrations of active compounds.

### 3.6. Antimicrobial Activity of Red Clover Extracts

The samples’ antimicrobial activities were investigated based on their diffusion into solid nutrition agar. The samples prepared with excipients (PVP/VAC and SSG in water or ethanol) and the controls (prepared without excipients in water or ethanol) were investigated against pathogenic bacteria. Still, there was no significant difference between the test samples and controls without excipients.

Comparing the samples prepared using ethanol and water, differences between their inhibition were found. The water samples inhibited five microorganisms, and the ethanol samples inhibited four (Table 4). Although the maximum concentration used in this study of extracts was 210 mg/mL, this concentration did not inhibit *Enterococcus faecalis*, *Pseudomonas aeruginosa*, or *Klebsiella pneumoniae* in both the water and ethanolic samples. Additionally, the ethanolic extract did not inhibit *Bacillus cereus.* Nevertheless, the ethanolic samples inhibited microorganisms in much lower concentrations than the aqueous samples. Generally, Gram-positive bacteria were more sensitive to the aqueous extracts tested than Gram-negative bacteria. However, the ethanolic extract had a similar effect on Gram-positive and Gram-negative bacteria.

The antimicrobial activity was assessed against key antibiotics, including penicillin, ciprofloxacin, ampicillin, amoxicillin, and daptomycin, targeting Gram-positive and Gram-negative bacteria. Penicillin exhibited potent activity against all Gram-positive bacteria tested, including *Staphylococcus aureus* (MIC > 0.25 μg/mL), *Staphylococcus epidermis* (MIC > 0.25 μg/mL), and *Enterococcus faecalis* (MIC > 16 μg/mL). Ciprofloxacin, while effective, required higher concentrations compared to penicillin for *Staphylococcus aureus* (MIC > 2 μg/mL), *Staphylococcus epidermis* (MIC > 2 μg/mL), and *Enterococcus faecalis* (MIC > 4 μg/mL). Daptomycin showed no inhibition for *Enterococcus faecalis*, and its MIC for *Staphylococcus aureus* and *Staphylococcus epidermis* exceeded 1 μg/mL.

Ampicillin and amoxicillin were tested against Gram-negative bacteria. Ampicillin failed to inhibit *Pseudomonas aeruginosa*. Against *Escherichia coli*, ampicillin had an MIC value of >32 μg/mL, and for *Klebsiella pneumonia*, it was ≥32 μg/mL. Amoxicillin did not inhibit *Klebsiella pneumonia* or *Pseudomonas aeruginosa* but demonstrated inhibitory activity against *Escherichia coli* with a MIC value of ≥32 μg/mL.

Despite certain antibiotics demonstrating higher inhibitory concentrations, this study underscores the considerable potential of red clover flower extracts. The results suggest that red clover extract, whether ethanolic or aqueous, should be employed in concentrated formulations to optimize its effects. This finding advocates for the strategic utilization of red clover in product development, emphasizing its unique attributes in achieving optimal therapeutic outcomes. Even though *Fabaceae* is the largest family with about 19.580 species, only 93 individual species of Fabaceae were reported for their antibacterial activities. This represents a relatively small percentage (0.5%) of the total diversity in this family [63]. Nevertheless, it was reported that isoflavone-containing plants like soybeans and alfalfa possess antioxidant activities. These plants especially inhibit *Escherichia coli* and *Staphylococcus aureus* [64,65].

The main isoflavone compounds found in red clover are daidzein and genistein. In the literature, it was reported that the metabolites of isoflavones can be even more potent antibacterial agents than their precursors. However, the same precursors (daidzein and genistein) possess antimicrobial activity, especially against Gram-positive bacteria and can act synergistically with other phenolic compounds in order to downregulate the resistance mechanisms of bacteria [66,67,68].

Even though there are limited studies regarding the antimicrobial effects of red clover flowers, some suggest that essential oil and extract do not possess antimicrobial and antifungal properties [69,70]. Nevertheless, this can be due to ineffective extraction methods. Gligor et al. [71] concluded that the samples’ biological activity depends on the phytochemical profile determined by the extraction technology.

## 4. Conclusions

The extract of red clover flowers is a promising natural alternative for the development of pharmaceuticals due to its high levels of phenolics and flavonoids as well as its antioxidant and antibacterial properties. The addition of the excipients SSG and PVP/VAC demonstrated a significant increase in the phenolic and flavonoid content in the extraction media, which enhanced their antioxidant activity. Although there was no difference in antimicrobial activity between the extracts with or without excipients, there were differences in the extraction solvents. Using excipients can safely enrich the extract with valuable compounds, particularly the PVP/VAC excipient, which showed greater results in both water and ethanol than the SSG excipient.

The extracts prepared using 5% PVP/VAC in water, as opposed to 1%, exhibited notably superior results across all the test samples. Specifically, sample V5W3 demonstrated the highest DPPH (11.25 ± 0.11 µg TE/g dw), ABTS (419.29 ± 7.00 µg TE/g dw) radical scavenging activity, and FRAP (167.45 ± 2.06 mg FS/g dw) reducing power activity among the aqueous samples. Additionally, V5W3 displayed the highest concentrations of phenols and flavonoid contents among the aqueous samples (45.88 ± 0.19 mg GAE/g dw and 23.10 ± 0.10 mg RU/g dw).

Regarding the ethanolic samples, the highest activity was observed in sample V1E3, boasting the highest phenol content (52.48 ± 0.24 mg GAE/g dw), flavonoid content (26.98 ± 0.28 mg RU/g dw), and antioxidant activity, including DPPH (12.81 ± 0.05 µg TE/g dw), ABTS (463.92 ± 6.46 µg TE/g dw), and FRAP (192.49 ± 1.45 mg FS/g dw. In the future, further research is needed to explore the potential applications of red clover flower extract in various pharmaceutical products.

## Figures and Tables

**Table 1:** DPPH and ABTS radical scavenging activity of extracts with different excipients, *n* = 4 (the codes of the samples are provided in Figure 1).

	DPPH, (µg TE/g dw) *	SD, *n* = 4	ABTS, (µg TE/g dw) *	SD, *n* = 4
W1	9.76	0.39	353.45	9.19
W2	9.04	0.22	348.14	4.18
W3	8.52	0.78	344.28	7.06
S1W1	10.50	0.15	389.06	6.45
S1W2	10.29	0.08	383.63	7.73
S1W3	10.17	0.02	388.60	6.40
V1W1	10.67	0.04	395.45	2.51
V1W2	10.98	0.10	397.70	6.54
V1W3	10.76	0.01	405.61	2.54
V5W1	10.76	0.06	402.55	8.12
V5W2	11.10	0.08	405.49	7.57
V5W3	11.25	0.11	419.29	7.00
E1	10.98	0.29	393.05	5.82
E1	10.73	0.25	390.39	7.41
E3	10.49	0.22	385.56	6.51
S1E1	11.84	0.11	433.48	3.84
S1E2	11.26	0.06	425.04	4.38
S1E3	12.02	0.13	436.06	8.17
V1E1	11.92	0.32	447.68	4.46
V1E2	12.25	0.10	460.67	5.08
V1E3	12.81	0.05	463.92	6.46

* All samples had statistically significantly higher antioxidant activity than the controls (*p* < 0.05).

**Table 2:** ABTS radical scavenging activity of extracts with different excipients, *n* = 4 (the codes of the samples are provided in Figure 1).

Samples Codes	ABTS Radical Scavenging, mg TE/g dw
W3	4.00 ± 0.16
S1W3	15.06 ± 0.60 ^b^
V1W3	20.90 ± 0.83 ^b^
E3	11.46 ± 0.45
S1E3	16.91 ± 0.83 ^a^
V1E3	29.04 ± 1.16 ^a^

^a^ *p* < 0.05 vs. E3; ^b^ *p* < 0.05 vs. W3. The control in water is compared only with samples prepared in water. Moreover, the controls prepared in ethanol are compared only with the ethanol samples.

**Table 3:** Identified compound concentrations expressed as hyperoside equivalent µg/mL.

Compounds nr.	W3, µg/mL	S1W3, µg/mL	V1W3, µg/mL	E3, µg/mL	S1E3, µg/mL	V1E3, µg/mL
**1**	19.25 ± 0.58	1.94 ± 0.058	2.7 ± 0.081	78.06 ± 2.34	25.81 ± 0.77	75.41 ± 2.26
**2**	36.33 ± 1.09	19.42 ± 0.58	49.41 ± 1.48	118.55 ± 3.56	38.77 ± 1.16	69.92 ± 2.10
**3**	77.3 ± 2.32	72.73 ± 2.18	25.71 ± 0.77	45.29 ± 1.36	97.64 ± 2.93	56.97 ± 1.71
**4**	18.91 ± 0.57	15.18 ± 0.46	16.61 ± 0.50	45.18 ± 1.36	88.5 ± 2.66	40.31 ± 1.21
**5**	46.13 ± 1.38	61.94 ± 1.86	45.16 ± 1.35	48.28 ± 1.45	75.66 ± 2.27	62.76 ± 1.88
**6**	10.3 ± 0.31	13.53 ± 0.41	3.75 ± 0.11	4.53 ± 0.14	10.3 ± 0.31	6.52 ± 0.20
**7**	17.65 ± 0.53	15.55 ± 0.47	23.42 ± 0.70	41.68 ± 1.25	46.13 ± 1.39	54.83 ± 1.65
**8**	15.83 ± 0.47	7.11 ± 0.21	0.28 ± 0.01	1.15 ± 0.04	44.19 ± 1.33	1.29 ± 0.04
**9**	0.33 ± 0.01	3.22 ± 0.10	0.79 ± 0.02	7.74 ± 0.23	73.02 ± 2.19	8.71 ± 0.26

**Table 4:** The antimicrobial activity (MIC) of red clover aqueous and ethanolic extracts, *n* = 3.

Solvent of Sample **	Gram-Positive Bacteria				Gram-Negative Bacteria			Yeast
	*Staphylococcus aureus* ATCC 25923	*Staphylococcus epidermidis* ATCC 12228	*Enterococcus faecalis* ATCC 29212	*Bacillus cereus* ATCC 11778	*Escherichia coli* ATCC 25922	*Pseudomonas* *aeruginosa* ATCC 27853	*Klebsiella pneumoniae* ATCC 13883	*Candida albicans* ATCC 10231
Water	125.51 mg/mL	65.92 mg/mL	NA	99.34 mg/mL	99.34 mg/mL	NA	NA	210.16 mg/mL
Ethanol	34.23 mg/mL	25.92 mg/mL	NA	NA mg/mL	34.23 mg/mL	NA	NA	34.23 mg/mL
Penicillin	>0.25 μg/mL	>0.25 μg/mL	>16 μg/mL	-	-	-	-	-
Ciprofloxacin	>2 μg/mL	>2 μg/mL	>4 μg/mL	-	-	-	-	-
Ampicillin	-	-	-	-	>61.9 μg/mL	NA	>32 μg/mL	-
Amoxicillin,	-	-	-	-	>32 μg/mL	NA	NA	-
Daptomycin	>1 μg/mL	>1 μg/mL	NA	-	-	-	-	-

NA means “no activity”. The extracts did not inhibit the reference microbial strains in their ≤ 210 mg/mL concentration. - means it was not tested with the bacteria. ** The antimicrobial activity was evaluated against both solvents. The solvents (water and 50% ethanol) alone did not inhibit the reference microbial strains.

## Data Availability

The data are contained within this article.
